# Magnetic Array Assisted Triboelectric Nanogenerator Sensor for Real-Time Gesture Interaction

**DOI:** 10.1007/s40820-020-00575-2

**Published:** 2021-01-05

**Authors:** Ken Qin, Chen Chen, Xianjie Pu, Qian Tang, Wencong He, Yike Liu, Qixuan Zeng, Guanlin Liu, Hengyu Guo, Chenguo Hu

**Affiliations:** 1 Department of Applied Physics, State Key Laboratory of Power Transmission Equipment & System Security and New Technology, Chongqing Key Laboratory of Soft Condensed Matter Physics and Smart Materials, Chongqing University, Chongqing, 400044 People’s Republic of China; 2 Center On Nanoenergy Research, School of Physical Science and Technology, Guangxi University, Nanning, Guangxi 530004 People’s Republic of China

**Keywords:** Sliding triboelectric sensor, Magnetic array, Gesture, Real-time, Human-machine interaction

## Abstract

In human-machine interaction, robotic hands are useful in many scenarios. To operate robotic hands via gestures instead of handles will greatly improve the convenience and intuition of human-machine interaction. Here, we present a magnetic array assisted sliding triboelectric sensor for achieving a real-time gesture interaction between a human hand and robotic hand. With a finger’s traction movement of flexion or extension, the sensor can induce positive/negative pulse signals. Through counting the pulses in unit time, the degree, speed, and direction of finger motion can be judged in real-time. The magnetic array plays an important role in generating the quantifiable pulses. The designed two parts of magnetic array can transform sliding motion into contact-separation and constrain the sliding pathway, respectively, thus improve the durability, low speed signal amplitude, and stability of the system. This direct quantization approach and optimization of wearable gesture sensor provide a new strategy for achieving a natural, intuitive, and real-time human-robotic interaction.

**Supplementary information:**

The online version contains supplementary material at (doi:10.1007/s40820-020-00575-2).

## Highlights

- By counting the positive/negative pulses in unit time to sense the degree, speed, and direction of finger motion in real-time.
- The magnetic array assisted sliding structure translates sliding motion into contact-separation thus improves the durability and low-speed signal amplitude.
- The magnetic track constrains the sliding direction that greatly improves the stability.

**Supplementary information:**

The online version contains supplementary material at (doi:10.1007/s40820-020-00575-2).

## Graphical abstract

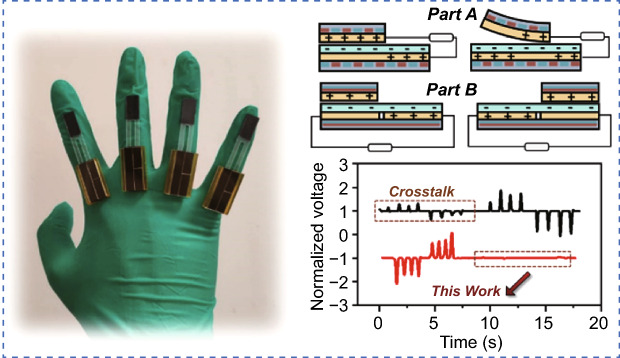

## Introduction

Intuitive human-machine interaction (HMI) plays more and more an important role in duties and daily life. From traditional keyboards to various touchpad nowadays, HMIs have been developed to be more natural [1], integrated [2], portable [3, 4], and even wearable [5, 6]. For the skillful robotic hand in industry, military service, surgery, or entertainment, traditional interaction method based on handles cannot meet current requirements [7, 8]. A wearable gesture sensor that can perceive the dexterous movement of each finger will fulfil this need [9–12]. The emergent triboelectric nanogenerator (TENG) exhibits great potential as an alternative scheme in some fields, including physiological signal collection [13, 14], mechanical signal detecting [15, 16], and visual tracking [8, 17]. Based on the coupling of triboelectrification and electrostatic induction, TENG has been applied in energy harvesting [18, 25, 26] and self-powered mechanical sensing [1, 19–24]. However, most studies in gesture sensing based on TENG take signal amplitude as feature to represent finger’s once time movement and ignore the intermediate process [13, 14, 16]. Moreover, to quantify the bending degree via signal amplitude is unstable, because there are too many factors affecting the amplitude. Therefore, a finger-wearable TENG sensor for quantifying the finger’s bending/straightening for a real-time gesture interaction is expected [15, 27].

In this work, we present a magnetic array assisted sliding triboelectric sensor (Ma-s-TS) to achieve a real-time gesture interaction between a human hand and robotic hand. During the bending of the finger, as shown in Fig. S1, the rotation angle is proportional to the tensile displacement at the joint (as the fulcrum). Therefore, We can judge the bending degree by the sliding displacement. The basic sensing principle of Ma-s-TS is to induce positive/negative pulses under the finger traction movement (flexion/extension), and then by counting the pulses in unit time, to sense the degree, speed and direction of a finger motion in real-time. It is a creative design that the magnetic array assisted sliding structure can constrain the sliding pathway and translate the sliding motion into contact-separation, and thus can improve the stability, durability and low speed signal amplitude. This work brings an optimized scheme for real-time gesture interaction based on a wearable TENG sensor, and it promises a widespread application of intuitive, natural HMIs.

## Methods

### Fabrication of Ma-s-TS

Typically, the Ma-s-TS was fabricated as two main parts, stator and slider. The stator (20 × 12 mm^2^) was made up of an electrode layer (thickness: 0.01 mm) sandwiched between upper layer of polytetrafluoroethylene (PTFE) film (thickness: 0.08 mm) and bottom layer of flexible halbach magnetic arrays made of strontium ferrite magnetic powder and rubber (thickness: 0.315 mm), which was pasted on an acrylic board. The slider (length: 10 mm, width: 20 mm) was a Y-shaped PET strip, which covered a layer of flexible rubber and a layer of halbach magnetic arrays. Finally, a copper layer (0.02 mm) was pasted on the surface of the as-fabricated flexible rubber as the positive tribo-material by polyimide tape. It should be noted that both the magnetic rubbers on the stator and slider were divided into two parts (part I and part II). The magnetic array of part I is perpendicular to the sliding direction, while that of Part II is parallel to the sliding direction, by which the two tribo-layers were alternately pushed up and down in sliding process.

### Characterization and Measurement

For electric signal test of the Ma-s-TS, a home-made hinge component [15] was fabricated to simulate the finger’s motion. A numerical controlled electric stepping motor was adopted to operate the Ma-s-TS. An electrometer (Keithley 6514) was used to measure the voltage signal. NI USB-6356 (National Instruments Corporation) was used for multi-channel data collection. The software was constructed on LabVIEW platform for real-time data acquisition, analysis and control. A commercial robotic hand (LOBOT uhand, Shenzhen Hiwonder Technology Co., Ltd.) was adopted in the real-time interaction system.

## Results and Discussion

**Fig. 1:**
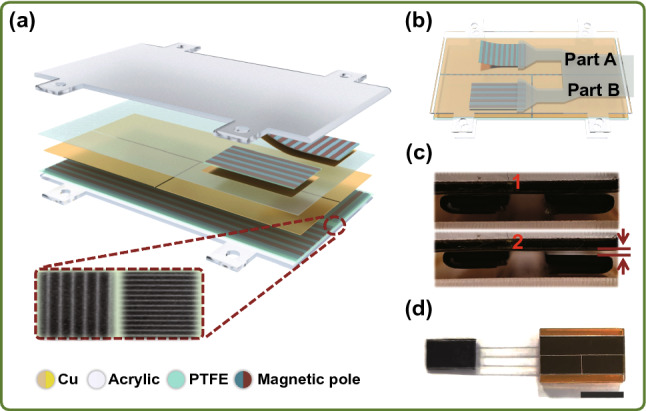
Structure of the Ma-s-TS. **a** Schematic diagram and multilayer structure of the Ma-s-TS. The insets: developing photograph of the magnetic array on the substrate and slider. **b** Overall structure diagram. **c** Magnetic array assisted contact-separation state (top and bottom). **d** Photograph of an as-fabricated sensor (scale bar: 1 cm)

**Fig. 2:**
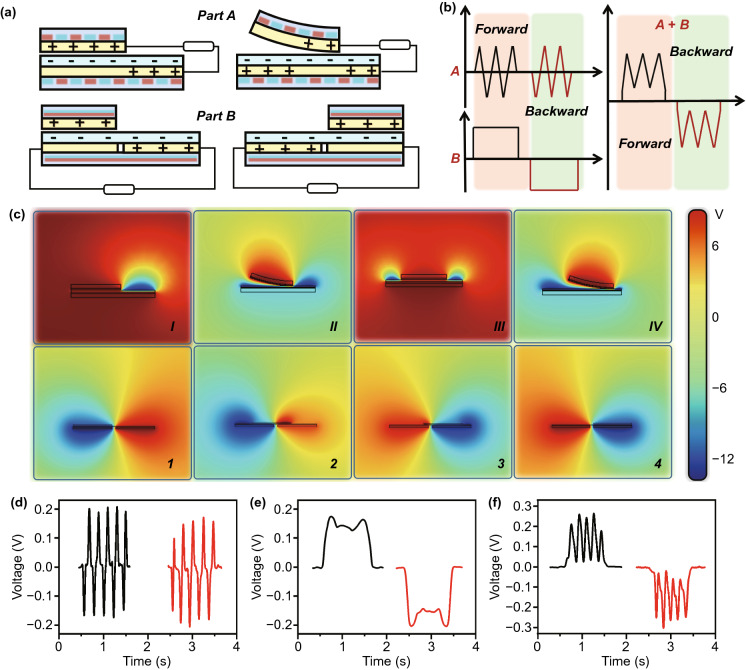
Operating-principle of the Ma-s-TS. **a** Schematics of electron transfer process in the sliding. **b** Schematics of output signal in sliding process. **c** COSMOL simulation of potential distribution under various states in sliding process. **d–f** Test output signal corresponding to Fig. 2b

The structure follows a sliding mode as illustrated in Fig. 1. Figure 1a presents the structural scheme of the Ma-s-TS, which consists of an acrylic rectangular cavity and a slider. The multilayer structure from bottom to top is the acrylic substrate, the magnetic stripe distributed in two vertical directions (part I and part II) in plane, the copper electrode divided into two parts, and the polytetrafluoroethylene (PTFE) surface. The slider also consists of two parts, which from bottom to top is the copper layer and the magnetic stripe distributed in same direction with the stator on each part. The insets show the developing photograph of the magnetic array on the substrate and slider. Left part forms an attractive track for stable sliding, while the right part forms the attraction–repulsion of magnetic pole in sliding. The operating principle is described later in Fig. 2. Figure 1b shows the overall structure diagram. Figure 1c shows the contact-separation state of the right part of slider and the stator in sliding process. In this process, the left part of slider is always in attraction state to the stator to form a stable sliding track. Figure 1d shows the as-fabricated Ma-s-TS. The detailed fabrication process is presented in the Experimental Section.

The operating mechanism of the Ma-s-TS is illustrated in Fig. 2. Firstly, electrons transfer from the electrode layer to the FEP film owing to the different triboelectric polarities. And then, for part A, the slider separates from the stator under the repulsive force of the magnetic field when their magnetic properties are identical. Keeping sliding, the slider contacts with the stator under the attraction when their magnetic properties are different. At the same time, for part B, the slider is always attracted to the stator and slides along a straight line due to the magnetic pull. Thus, as shown in Fig. 2a, b, the alternating electric signals from the paired double electrodes in contact-separation state are a series of periodic narrow pulses in part A. For part B, the electrode in free-standing mode generates alternating wide-pulse signals in a sliding cycle. These two kinds of signals are coupled to form a series of positive/negative pulses representing the finger’s flexion/extension. To interpret the working principle, the potential distribution of part A electrode (top, Fig. 2c) and part B electrode (bottom, Fig. 2c) under open-circuit condition is simulated by COMSOL in four phases of an ordinary cycle. The actual measured open-circuit voltage of part A and part B and that of the coupled circuit can be referred to Fig. S2. Furthermore, corresponding to Fig. 2b, the load voltage signals out of part A and part B are individually measured to verify the working principle. Figure 2d–f shows the real signals detected from the two independent parts and the coupled circuit.

**Fig. 3:**
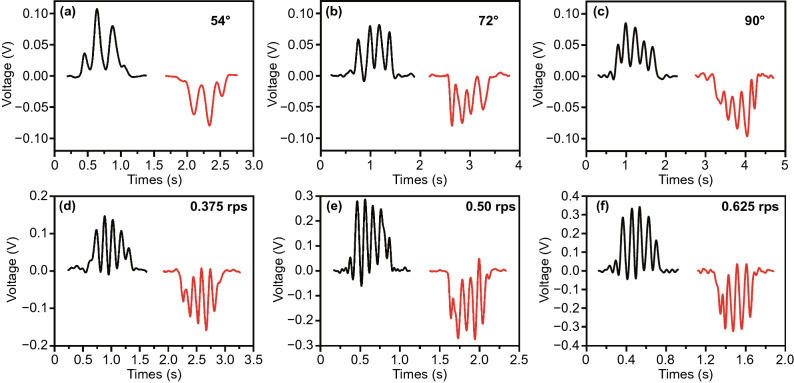
Characterization of the basic performance of Ma-s-TS. **a–c** Pulses produced from a Ma-s-TS when rotate through different angles (54°, 72°, and 90°) at rotation speed of 0.375 rps. **d–f** The pulses generated from a Ma-s-TS when rotate through 90° at different rotation speed (0.375, 0.50, and 0.625 rps)

To quantitatively characterize the performance of the Ma-s-TS, a numerical controlled electric stepping motor is used to drive a hinge component to operate it [15]. To demonstrate the pulse number representing the finger’s flexion/extension degree, the stepping motor is set to rotate through different angles (54°, 72°, and 90°) at a rotation speed of 0.375 rps. Meanwhile, the sequences of pulses generated by the Ma-s-TS are recorded, as illustrated in Fig. 3a–c, respectively. The generated pulse number is linear to the rotation angle. Therefore, the pulse number represents the finger’s flexion/extension degree. To investigate the influence of rotation speed, the stepping motor is set to rotate through 90° at different rotation speed (0.375, 0.50, and 0.625 rps). From Fig. 3d–f, it can be found that the total pulse number is a constant at the same rotation degree, which means through counting the pulse number the finger’s flexion/extension degree can be judged stably/accurately. In addition, through calculating the number of pulses per unit time, the motion speed can also be determined. The influence of the thickness to sensing accuracy is also tested in Fig. S3, because different thickness of magnetic stripe has different number of pole pairs. Meanwhile, the relationship between driving force and thickness also be explored in Fig. S4.

**Fig. 4:**
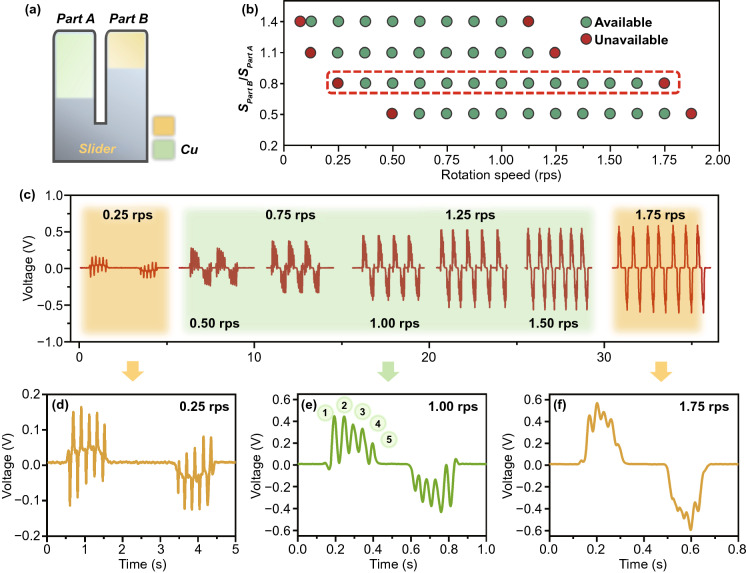
Matching of two coupled parts of the slider. **a** Area adjustment diagram of the two parts. **b** Speed response range under different area ratio of part B to part A. **c** Test signals at rotation speed from 0.25 to 1.75 rps under area ratio of 0.8. **d–f** Enlarged signal of Fig. 4c at rotation speed of 0.25, 1.00, and 1.75 rps

The output signal is a coupled signal of two parts of electrode. Therefore, the area ratio of the two parts will affect the character of the output signal, hence further affect the back-end interaction. The detailed features are studied as shown in Fig. 4. Figure 4a illustrates the area adjustment diagram of the copper electrode of part A and part B, which corresponds to narrow pulses and wide pulses, respectively. According to the test data, as shown in Fig. 4b, when area ratio of part B to part A increases, the motion speed response range shifts left. To further understand this feature, the test data of an area ratio of 0.8 is illustrated in Fig. 4c. Figure 4d–f is the enlarged signals of Fig. 4c at a low, medium, and high rotation speed for a better understanding. From rotation speed of 0.25–1.75 rps, when the slider moves slowly, the positive/negative narrow pulses are almost evenly distributed beside baseline and not be biased by the wide pulse from part B, hence hard to be distinguished (as shown in Fig. 4d). When the slider moves fast, the positive/negative narrow pulses are excessively biased by the wide pulse from part B and are hardly distinguished (as shown in Fig. 4f). For different response range requirements, in the state of slow sliding, a bigger area ratio of part B to part A helps getting a bigger bias (left shifts, Fig. 4b); while in the state of fast sliding, a smaller area ratio of part B to part A helps getting a smaller bias (right shifts, Fig. 4b). An ideal state in Fig. 4e shows that the best response range of the area ratio of 0.8 is around 1.0 rps. Therefore, it is necessary to choose the area ratio of the two parts of Ma-s-TS according to the speed response range requirements in different applications.

**Fig. 5:**
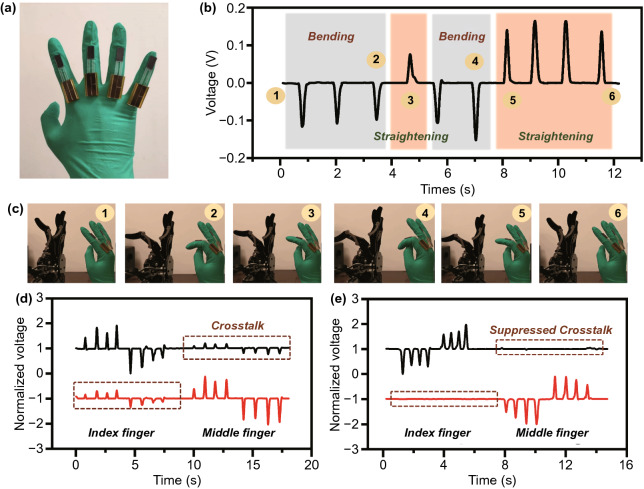
Real-time gesture interaction demonstration. **a** Ma-s-TS worn on the fingers. **b** Positive and negative pulses represent straightening and bending of finger. **c** Real-time gesture interaction of robotic hand and human hand based on the signal of Fig. 5b. **d–e** Comparison with jmTQS [15] on cross talk between channels (**d** jmTQS, **e** Ma-s-TS)

Based on the features of the Ma-s-TS above, a real-time gesture interaction system is demonstrated as shown in Fig. 5. Figure 5a shows the Ma-s-TS worn on the fingers. In Fig. 5b, the positive/negative pulses represent extending and bending of fingers, respectively. The phases of the human-robotic hand corresponding to the mark numbers in Fig. 5b are demonstrated in Fig. 5c. The motion of the robotic finger is totally/completely synchronous to the motion of human fingers in real-time. A real-time multi-directional continuous control of the robotic hand is demonstrated in Video S1. In this demonstrations, the real-time control can recover at any breakpoint in finger’s flexion/extension process. Besides, Ma-s-TS also showed good stability in multichannel control. When some fingers bend, the other fingers are often driven unconsciously. And the unconscious movement will always produce a wrong cross talk signal. As demonstrated in Video S2, Ma-s-TS can suppressed the cross talk between different channels effectively, benefitting from restriction of magnetic force to slider. Here, the cross talk between channels of the Ma-s-TS (Fig. 5e) is compared with previous joint motion triboelectric quantization sensor (Fig. 5d) [15]. The Ma-s-TS is more stable and thus the cross talk caused by finger linkage is suppressed, which is very important for achieving a stable real-time gesture interaction. Futhermore, the durability of Ma-s-TS also has been tested in Fig. S5. After 6,200 operation cycle (at the working frequency of 0.5 Hz), the result shows that the normalized output declines to 84.6% due to intermittent contact. Besides, the output signal of previous work produced continuous narrow pulse signals based on the freestanding model is greatly affected after working for a long time. Hence, we also compared the durability of part A with previous work, as shown in Fig. S5b.

## Conclusions

In summary, a magnetic array assisted sliding triboelectric sensor has been proposed for achieving a real-time gesture interaction between a human hand and robotic hand. Through counting the positive/negative pulses that represent extending and bending of fingers, respectively, the degree, speed and direction of the fingers’ flexion/extension can be judged. Besides, the magnetic array assisted sliding structure constrains the sliding direction and translates the sliding motion into contact-separation, which greatly improve the stability, durability and low-speed signal amplitude. Based on these novelties, a real-time gesture interaction system has been established. Furthermore, this Ma-s-TS can be applied in other similar joint’s motion detection for a more natural, high-precision and real-time synchronous human-machine interaction.

## Supplementary information

Supplementary file1 (PDF 462 kb)

Supplementary file2 (MP4 3964 kb)

Supplementary file3 (MP4 3815 kb)
