# Supplementary material for: Magnetic Array Assisted Triboelectric Nanogenerator Sensor for Real-Time Gesture Interaction
Source: Nanomicro Lett. 2021 Jan 5;13:51. doi: 10.1007/s40820-020-00575-2 (PMC8187499; doi:10.1007/s40820-020-00575-2)
Supplement: Supplementary file 1 — Supplementary file1 (PDF 462 kb) [file 40820_2020_575_MOESM1_ESM.pdf]

Supporting Information for

Magnetic Array Assisted Triboelectric Nanogenerator Sensor for Real-Time Gesture Interaction

Ken Qin[1, †], Chen Chen[1, †], Xianjie Pu[1, *], Qian Tang[1, *], Wencong He[1], Yike Liu[1], Qixuan Zeng[1], Guanlin Liu[2], Hengyu Guo[1], Chenguo Hu[1, *]

[1]Department of Applied Physics, State Key Laboratory of Power Transmission Equipment & System Security and New Technology, Chongqing Key Laboratory of Soft Condensed Matter Physics and Smart Materials, Chongqing University, Chongqing 400044, P. R. China

[2]Center on Nanoenergy Research, School of Physical Science and Technology, Guangxi University, Nanning, Guangxi 530004, P. R. China

[†]Ken Qin and Chen Chen contributed equally to this work

*Corresponding authors. E-mail: xjpu@cqu.edu.cn (X. Pu); tangqian@cqu.edu.cn (Q. Tang); hucg@cqu.edu.cn (C. Hu)

Supplementary Figures

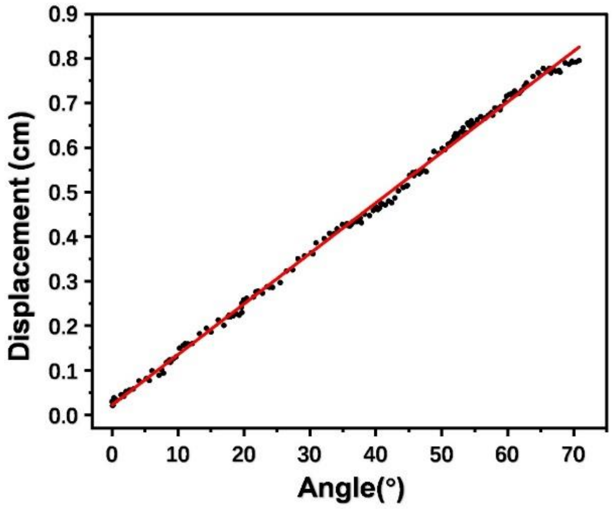

Fig. S1 Relationship between the angles of joint bending and the sliding displacement

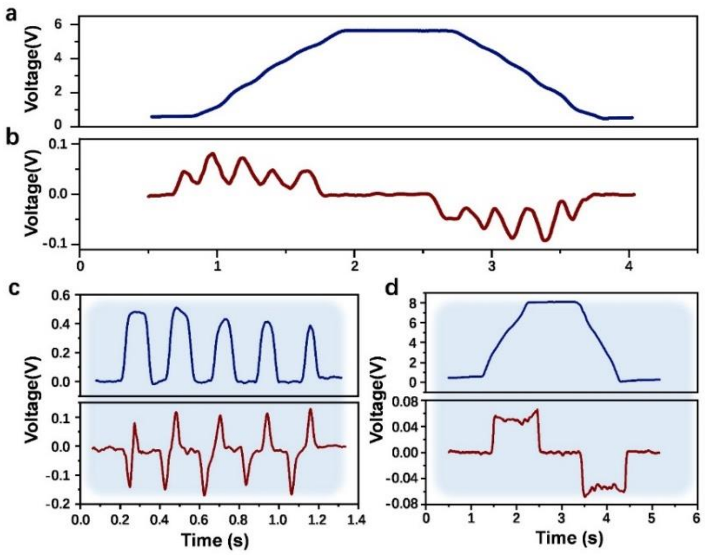

Fig. S2 Open circuit voltage and load voltage (20MΩ) of Ma-s-TS. (a-b) Open circuit voltage and load voltage of the coupled two parts of Ma-s-TS. **(c)** Open circuit voltage (up) and load voltage (down) of part A in half a cycle. **(d)** Open circuit voltage (up) and load voltage (down) of Part B in a full cycle

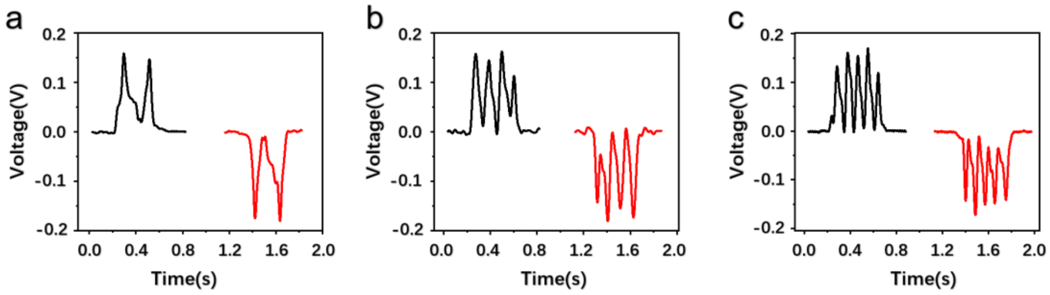

Fig. S3 Accuracy affected by the thickness of magnetic stripe. The pulses produced from the Ma-s-TS adopting magnetic stripe with different thickness (**(a)** 1.000 mm, **(b)** 0.500 mm and **(c)** 0.315 mm) when rotating through the same degree at a certain rotation speed

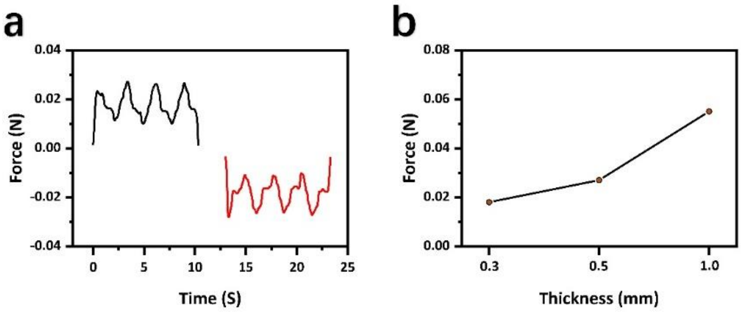

Fig. S4 Relationship between driven force and thickness (a) the force-time curve of the magnetic stripe thickness of 0.5 mm. **(b)** Minimum driving force under different magnetic stripe thicknesses (0.3, 0.5, and 1.0 mm)

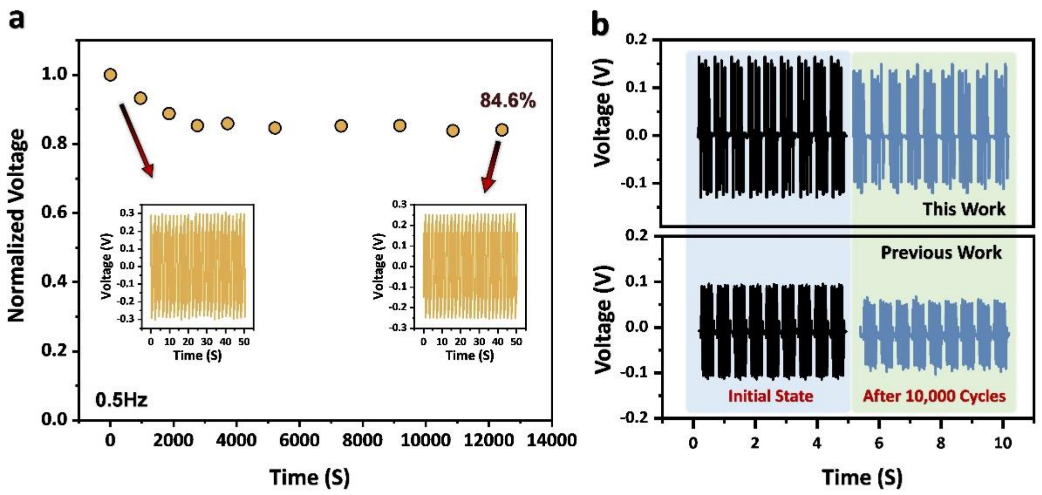

Fig. S5 Durability of Ma-s-TS. (**a**) After 6,200 continuous operation cycles, the normalized output of Ma-s-TS declined to 84.6%. (**b**) After 10,000 cycles, the durability of the narrow pulse segment is compared between this work and precious work
